# AI-Enabled Sensor Technologies for Remote Arrhythmic Monitoring in High-Risk Cardiomyopathy Genotypes

**DOI:** 10.3390/s26072078

**Published:** 2026-03-26

**Authors:** Nardi Tetaj, Andrea Segreti, Francesco Piccirillo, Aurora Ferro, Virginia Ligorio, Alberto Spagnolo, Michele Pelullo, Simone Pasquale Crispino, Francesco Grigioni

**Affiliations:** 1 Cardiology Unit, Policlinico Universitario Campus Bio-Medico, Via Alvaro del Portillo 200, 00128 Rome, Italy; nardi.tetaj@unicampus.it (N.T.); f.piccirillo@unicampus.it (F.P.); virginia.ligorio@unicampus.it (V.L.); alberto.spagnolo@unicampus.it (A.S.); michele.pelullo@unicampus.it (M.P.); simone.crispino@poclinicocampus.it (S.P.C.); f.grigioni@poclinicocampus.it (F.G.); 2 Research Unit of Cardiovascular Science, Department of Medicine and Surgery, Università Campus Bio-Medico di Roma, Via Alvaro del Portillo 21, 00128 Rome, Italy

**Keywords:** genetic cardiomyopathy, *LMNA*, *FLNC*, *RBM20*, *PLN*, desmosomal genes, arrhythmias, wearable sensors, implantable devices, remote monitoring, artificial intelligence

## Abstract

Inherited cardiomyopathies associated with high-risk genotypes, are characterized by a disproportionate risk of malignant ventricular arrhythmias and sudden cardiac death, often independent of left ventricular systolic dysfunction or advanced structural remodeling. Traditional surveillance strategies based on intermittent electrocardiography and phenotype-driven risk assessment are insufficient to capture the dynamic and often silent progression of electrical instability in these populations. This narrative review evaluates the emerging role of artificial intelligence (AI)-enabled sensor technologies in remote arrhythmic monitoring of genetically defined cardiomyopathy cohorts. Wearable ECG devices, implantable cardiac monitors, multisensor cardiac implantable electronic device algorithms, pulmonary artery pressure sensors, and contact-free systems enable continuous acquisition of electrophysiological and hemodynamic data, generating digital biomarkers that may reflect early arrhythmic vulnerability and subclinical decompensation. AI-driven analytics enhance signal processing, automated event detection, and remote data triage, with the potential to reduce clinical workload while preserving diagnostic sensitivity. However, current evidence predominantly derives from heterogeneous heart failure or general arrhythmia populations, and prospective validation in genotype-specific cohorts remains limited. Key challenges include algorithm generalizability, signal quality in ambulatory environments, data governance, interpretability of AI models, and integration into structured remote-care pathways. The convergence of genotype-informed risk stratification and multimodal AI-enabled sensing represents a promising strategy to transition from reactive device-based protection to proactive, precision-guided arrhythmic prevention. Dedicated genotype-focused studies and standardized digital endpoints are required to support safe and effective implementation in inherited cardiomyopathies.

## 1. Introduction

In recent years, advances in cardiovascular genetics have demonstrated that specific inherited cardiomyopathy genotypes confer a disproportionately high risk of malignant ventricular arrhythmias and sudden cardiac death, often independent of left ventricular systolic function or overt structural disease [1]. Variants in *LMNA*, truncating *FLNC*, *RBM20*, *PLN* (p.Arg14del), and desmosomal genes exemplify this paradigm, challenging traditional phenotype-driven approaches to risk stratification and follow-up [2]. High-risk cardiomyopathy genotypes are characterized by distinct electrophysiological remodeling processes that can manifest as measurable physiological signals captured by modern sensor technologies. For example, *LMNA*-related cardiomyopathy frequently produces progressive conduction slowing and atrioventricular block that are detectable through prolonged PR interval changes or intermittent pauses on ambulatory ECG monitoring [3]. Similarly, truncating *FLNC* variants and *RBM20* mutations are associated with increased ventricular ectopy and non-sustained ventricular tachycardia, which may appear as dynamic changes in ectopic burden or ventricular arrhythmia episodes during continuous rhythm surveillance [4]. In desmosomal cardiomyopathies, exercise-triggered ventricular arrhythmias may be detected through wearable ECG systems combined with activity sensors [5]. These genotype-specific electrophysiological alterations therefore generate quantifiable physiological signals—such as rhythm disturbances, conduction interval changes, heart-rate variability alterations, and ectopic burden—that can be captured by wearable, implantable, or ambient sensors and subsequently analyzed using AI-enabled algorithms. Traditional approaches to arrhythmic surveillance in cardiomyopathy have relied on intermittent electrocardiography, short-term Holter monitoring, or symptom-triggered evaluations [6]. While these tools remain clinically valuable, they are intrinsically constrained by limited temporal resolution and poor alignment with the fluctuating nature of arrhythmic burden. As a result, clinically meaningful electrical instability may remain undetected until the occurrence of advanced heart failure (HF), sustained ventricular arrhythmias, or sudden cardiac death (SCD). Remote monitoring strategies, including wearable and implantable sensors, have therefore emerged as a complementary paradigm aimed at continuous or near-continuous physiological surveillance, with the potential to identify early markers of arrhythmic risk and clinical deterioration [7].

A growing body of evidence supports the feasibility of wearable, implantable, and contact-free sensors for long-term cardiovascular monitoring, including arrhythmia detection, HF surveillance, and physiological trend analysis [8]. Despite rapid technological progress, several critical gaps persist. Most AI-enabled monitoring systems have been developed and validated in heterogeneous cardiovascular populations. Prospective data linking sensor-derived digital biomarkers to arrhythmic outcomes in genetically defined cohorts are scarce, and the generalizability of AI models across rare variants remains uncertain. In the context of remote physiological monitoring, the term sensor-derived digital biomarkers refers to quantitative physiological indicators extracted from continuously acquired biosignals using digital sensors and computational analysis. Unlike traditional clinical biomarkers obtained through laboratory testing or intermittent clinical measurements, digital biomarkers are derived from longitudinal data streams such as electrocardiographic signals, photoplethysmography, activity metrics, or hemodynamic trends [9]. These metrics may include arrhythmia burden, heart rate variability patterns, conduction interval dynamics, or activity-adjusted rhythm instability. When analyzed longitudinally, such parameters may provide early indications of electrophysiological instability or clinical deterioration before conventional clinical markers become evident.

The present narrative review aims to synthesize current evidence on sensor-derived monitoring targets for remote arrhythmic monitoring and AI-based analytical frameworks, with a specific focus on high-risk cardiomyopathy genotypes.

## 2. Methods

**Figure 1:**
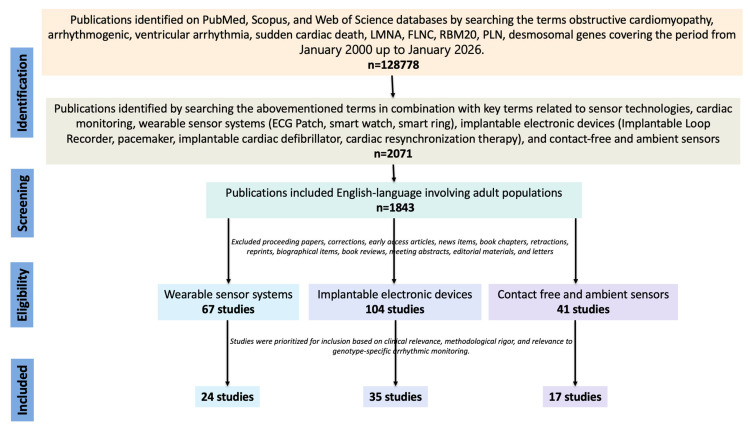
Literature search and study selection workflow for the narrative review.

A narrative literature search was conducted using PubMed, Scopus, and Web of Science databases to identify studies published between January 2000 up to January 2026. The search key strategy used the terms cardiomyopathy, arrhythmogenic, ventricular arrhythmia, sudden cardiac death, high-risk cardiomyopathy gene names (*LMNA, FLNC, RBM20, PLN,* desmosomal genes) combined with terms sensor technologies, cardiac monitoring, wearable sensor systems (ECG Patch, smart watch, smart ring), implantable electronic devices (Implantable Loop Recorder, pacemaker, implantable cardiac defibrillator, cardiac resynchronization therapy), and contact-free and ambient sensors. We included relevant English-language studies involving adult and pediatric populations, with a focus on cohorts and registries, risk-score derivation/validation studies, gene–disease validity papers, and ESC/consensus documents. We excluded proceeding papers, corrections, early access articles, news items, book chapters, retractions, reprints, biographical items, book reviews, meeting abstracts, editorial materials, and letters. This review was conducted as a structured narrative synthesis rather than a formal systematic review. Given the heterogeneity of sensor technologies and the limited genotype-specific validation studies, a qualitative integrative approach was adopted. Studies were prioritized for inclusion based on clinical relevance, methodological rigor, and relevance to genotype-specific arrhythmic monitoring. Prospective validation studies, device trials, and major guideline documents were preferentially considered, as shown in Figure 1. No formal meta-analysis or quantitative bias assessment was performed. The objective was to provide a translational framework linking genotype-informed arrhythmic risk with emerging AI-enabled monitoring strategies.

## 3. High-Risk Cardiomyopathy Genotypes and the Rationale for Intensive Monitoring

Growing evidence indicates that certain cardiomyopathy-associated genes define arrhythmogenic phenotypes characterized by early electrical instability, conduction disease, and sudden death risk that may precede advanced myocardial remodeling [10,11].

**Figure 2:**
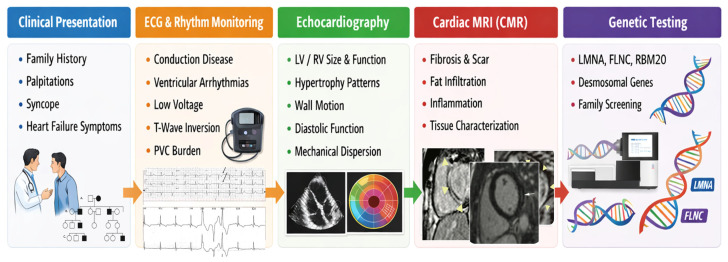
Initial diagnostic evaluation in patients with suspected cardiomyopathy. Abbreviations: LV, left ventricle; RV, right ventricle; PVC, premature ventricular contractions.

The high-risk cardiomyopathy genotypes include *LMNA, FLNC* truncating variants, *RBM20, PLN* (particularly *p.Arg14del*) and desmosomal genes (*DSP, PKP2, DSG2, DSC2, TMEM43*) [12,13]. Not all genotype-positive cardiomyopathy patients share the same trajectory. High-risk genotypes tend to combine: (i) malignant arrhythmic profiles (NSVT, sustained VT/VF, SCD); (ii) aggressive structural evolution often associated with myocardial fibrosis on CMR; and (iii) electrical or systemic red flags (conduction disease, low-voltage ECG, family clustering of early SCD or end-stage HF) [1,14,15]. Figure 2 shows a stepwise multimodal approach integrating clinical presentation, electrocardiographic assessment, echocardiographic structural evaluation, cardiac magnetic resonance tissue characterization, and genetic testing. The integration of electrical, structural, and molecular data enables etiologic diagnosis and personalized arrhythmic risk stratification.

Pathogenic variants in *LMNA* are associated with a cardiomyopathy phenotype marked by early atrioventricular conduction disease, atrial arrhythmias, non-sustained ventricular tachycardia, and a high incidence of life-threatening ventricular arrhythmias [16]. Importantly, arrhythmic events frequently occur in the setting of mildly reduced or even preserved left ventricular ejection fraction [17,18].

Truncating variants in *FLNC* define a malignant dilated or arrhythmogenic cardiomyopathy characterized by extensive myocardial fibrosis, frequent ventricular arrhythmias, and SCD often out of proportion to systolic dysfunction [19,20,21,22].

*RBM20* cardiomyopathy represents a prototypical example of a genotype associated with aggressive disease progression and early arrhythmic risk [23,24]. In many carriers, malignant ventricular arrhythmias develop at a young age, sometimes before overt dilation or HF symptoms, suggesting a window for pre-phenotypic electrical monitoring [25,26].

The *PLN p.Arg14del* variant is associated with a mixed dilated and arrhythmogenic phenotype characterized by low-voltage electrocardiograms, ventricular arrhythmias, and progressive HF [27]. Subtle changes in ECG amplitude, rhythm instability, or nocturnal arrhythmias may precede clinical deterioration [28,29].

Finally, desmosomal gene variants underpin arrhythmogenic cardiomyopathy, in which ventricular arrhythmias are often exercise-related and may occur in the absence of severe ventricular dysfunction [30]. The dynamic interaction between physical activity and electrical instability highlights the value of multimodal sensing that incorporates both rhythm and activity data [31,32]. Traditional episodic monitoring strategies lack temporal resolution to detect intermittent ventricular arrhythmias or progressive conduction abnormalities, particularly when asymptomatic [33,34].

Given the heterogeneity of arrhythmic trajectories across high-risk cardiomyopathy genotypes, a genotype-informed monitoring strategy is warranted. Table 1 proposes a genotype-informed framework aligning arrhythmic phenotype with monitoring intensity and sensor selection.

From a pathophysiological perspective, intensive monitoring in high-risk cardiomyopathy genotypes should not be conceptualized solely as arrhythmia detection, but rather as continuous surveillance of electrical instability and its physiological correlates. Changes in ventricular ectopic burden, heart rate (HR), heart rate variability (HRV), conduction intervals, activity patterns, and hemodynamic parameters may all serve as early indicators of arrhythmic vulnerability [40,41]. Multisensor monitoring systems and implantable or wearable technologies are uniquely positioned to capture these signals over extended periods, providing a more comprehensive representation of disease dynamics than isolated measurements. Intensive monitoring may therefore serve multiple complementary purposes: early identification of electrical instability, refinement of individual risk profiles over time, optimization of device therapy and pharmacological management, and improved timing of prophylactic interventions. However, the volume and complexity of data generated by continuous monitoring also introduce new challenges, underscoring the need for advanced analytical frameworks capable of extracting clinically meaningful information without overwhelming healthcare systems [42,43].

**Figure 3:**
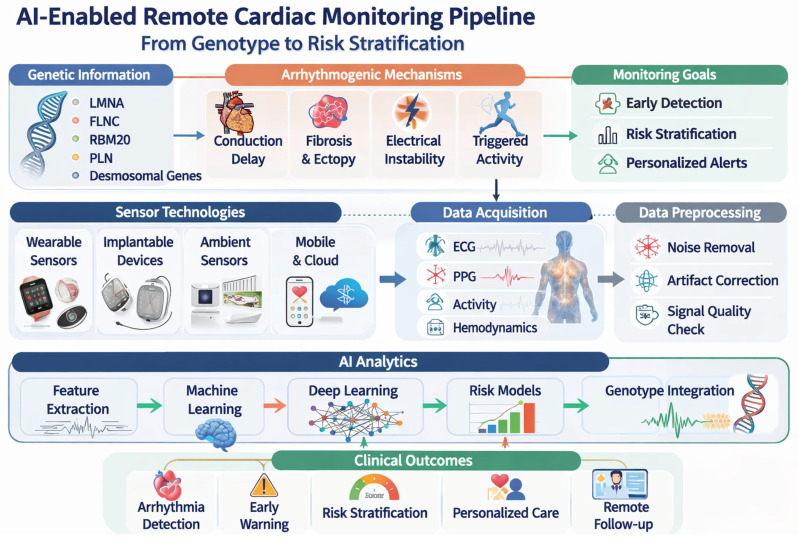
AI-enabled monitoring pipeline for genotype-informed arrhythmic surveillance. Physiological signals acquired from wearable, implantable, and ambient sensors undergo preprocessing and quality assessment before feature extraction and AI-based analysis. The resulting digital biomarkers may support arrhythmic risk stratification and clinical decision-making. Abbreviations: PPG, photoplethysmography.

Artificial intelligence-based approaches are increasingly viewed as a critical enabler of intensive monitoring in this context. By facilitating automated signal interpretation, temporal pattern recognition, and risk prioritization, AI has the potential to transform continuous data streams into actionable clinical insights [44]. This capability is especially pertinent in genetically mediated cardiomyopathies, where arrhythmic burden may be intermittent, and with rare events, heterogeneous phenotypes, and long observation periods complicate traditional analytical strategies. As such, the integration of AI-enabled analytics with sensor-based monitoring forms the conceptual foundation for genotype-informed arrhythmic surveillance, Figure 3. Artificial intelligence and advanced signal processing integrate longitudinal, multimodal data streams may support anticipatory arrhythmic risk assessment and personalized clinical decision-making.

In a subset of cardiomyopathy genotypes, arrhythmic risk is strongly modulated by physical activity and adrenergic stimulation. Ventricular arrhythmias may be rare at rest but become more frequent or malignant during exertion, emotional stress, or competitive sports [38,45]. Wearable sensors incorporating ECG, accelerometry, and activity tracking are particularly valuable in this setting, as they allow correlation of arrhythmic events with real-world physical activity patterns. Genotype-informed monitoring strategies in this group emphasize context-aware surveillance, where AI algorithms assess arrhythmic burden relative to activity intensity and recovery dynamics. This approach may support individualized recommendations regarding exercise participation and risk mitigation, while avoiding unnecessary restrictions based on isolated or decontextualized arrhythmic findings.

## 4. Sensor Technologies for Cardiac Monitoring

Sensor-based cardiac monitoring technologies constitute the technical foundation of remote arrhythmic surveillance in cardiomyopathy patients. Over the past decade, advances in miniaturization, wireless connectivity, and signal processing have enabled the deployment of a broad spectrum of sensors capable of capturing electrical, mechanical, and hemodynamic signals over prolonged periods. These technologies differ substantially in invasiveness, signal fidelity, monitoring duration, and clinical objectives, yet they share a common goal: to provide continuous or near-continuous insight into cardiovascular physiology beyond the constraints of traditional clinical encounters [46].

**Figure 4:**
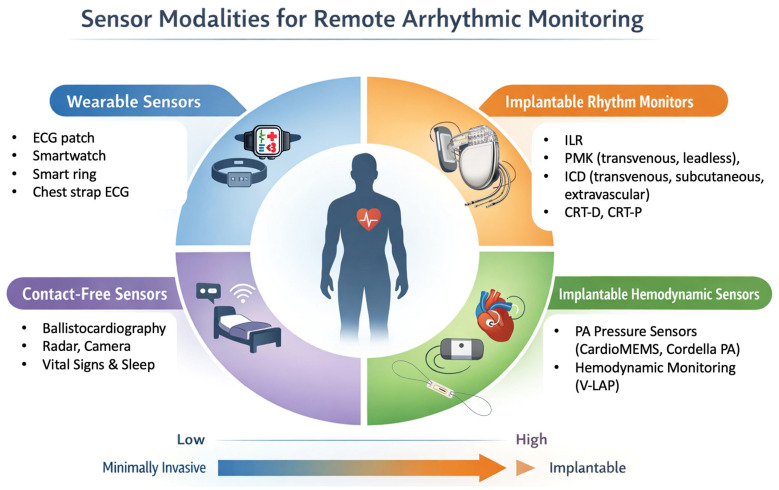
Schematic overview of current sensor technologies used for remote surveillance of arrhythmias and cardiovascular instability. Abbreviations: ILR, implantable loop recorder; PMK, pacemaker; ICD, implantable cardioverter defibrillator; CRT-D, cardiac resynchronization therapy defibrillator; CRT-P, cardiac resynchronization therapy pacemaker; PA, pulmonary pressure; V-LAP, vectorious left atrial pressure.

From a clinical perspective, sensor technologies for cardiac monitoring can be broadly categorized into wearable devices, implantable systems, and contact-free or ambient sensors, as shown in Figure 4. Each category offers distinct advantages and limitations, and their relevance varies according to arrhythmic phenotype, disease stage, and patient-specific risk profile.

### 4.1. Wearable Sensor Systems

Wearable sensors represent the most accessible and scalable approach to long-term cardiac monitoring. These devices typically incorporate surface electrocardiography (ECG), photoplethysmography (PPG), accelerometry, and, in some cases, additional physiological sensors to capture HR, HRV, physical activity, and sleep patterns [47]. Their non-invasive nature and widespread adoption have facilitated large-scale monitoring initiatives and real-world data generation. PPG is a non-invasive optical sensing technique that detects blood volume changes in the microvascular bed using light-emitting diodes and photodetectors. By measuring variations in light absorption associated with pulsatile arterial flow, PPG enables continuous assessment of HR and HR variability and can support algorithm-based detection of arrhythmias such as atrial fibrillation, and to indirectly estimate blood pressure (BP) [48]. Widely integrated into smartwatches, rings, and wearable pulse oximeters, PPG offers scalable and user-friendly cardiovascular monitoring.

In carriers of high-risk cardiomyopathy genotypes (e.g., *LMNA, FLNC, RBM20, PLN*), wearable monitoring strategies should reflect the anticipated arrhythmic phenotype and stage of disease expression. In early or genotype-positive/phenotype-negative individuals, ECG-enabled smartwatches may support intermittent rhythm screening and longitudinal autonomic profiling. However, their role remains adjunctive rather than diagnostic. In patients with cardiomyopathy family history, conduction disease, or increasing premature ventricular contraction burden, continuous ambulatory ECG patch monitoring (7–14 days) is preferable to detect non-sustained ventricular tachycardia (NSVT) and dynamic arrhythmic substrate progression [49,50]. For individuals with recurrent syncope, rapidly evolving conduction abnormalities, or documented NSVT, extended monitoring (up to 30 days via mobile cardiac telemetry) may provide higher diagnostic yield while informing timing of ICD implantation in accordance with ESC 2023 recommendations [1]. Table 2 shows a comprehensive overview of currently available wearable technologies for cardiovascular monitoring. Table 2, Table 3 and Table 4 summarize representative monitoring technologies currently available across wearable, implantable, and ambient sensor platforms, providing a consolidated overview of the rapidly evolving digital cardiology landscape.

However, in these patients, multimodal wearables integrating ECG, HRV, activity, and respiratory metrics may offer additional prognostic stratification, although they do not replace implantable rhythm surveillance when arrhythmic risk is substantial. Thus, wearable technologies should be viewed as complementary tools within a genotype-guided risk stratification framework rather than standalone decision-making instruments. Furthermore, wearable sensors are subject to several limitations that are particularly relevant in cardiomyopathy populations. Motion artifacts, variable signal quality during physical activity, and reduced specificity for ventricular arrhythmias may compromise diagnostic accuracy. Also, signal quality may be affected by skin tone variability, temperature and peripheral perfusion, in PPG devices. Adherence and long-term compliance can be inconsistent, potentially limiting their utility for sustained risk surveillance in selected high-risk individuals [43,51].

### 4.2. Implantable Electronic Devices

In patients carrying high-risk cardiomyopathy genotypes, such as *LMNA, FLNC, RBM20, PLN*, and desmosomal genes, implantable cardiac devices function not only as therapeutic tools but as continuous phenotyping platforms [10]. These genotypes are characterized by early conduction disease, progressive ventricular dysfunction, and increased arrhythmic susceptibility, often preceding overt structural remodeling [52]. Consequently, long-term, high-resolution sensing becomes central to risk stratification. Implantable cardiac devices have progressively evolved from purely therapeutic systems into long-term, autonomous sensing platforms capable of generating continuous electrophysiological and hemodynamic datasets. These devices integrate miniaturized biosensors, embedded signal-processing algorithms, low-power circuitry, and wireless telemetry, enabling remote cardiovascular surveillance over periods ranging from several years to a decade [53]. Electrophysiological sensing platforms include implantable loop recorders (ILRs), pacemakers (PMK), implantable cardioverter-defibrillators (ICD), and cardiac resynchronization therapy systems (CRT-P/CRT-D) [54]. Overview of currently available implantable cardiac devices are shown in Table 3. While implantable and wearable platforms are increasingly sophisticated, comparative performance metrics remain insufficiently standardized. Future studies should prioritize standardized digital endpoints to allow meaningful cross-platform comparison.

ILRs function as subcutaneous single-lead ECG acquisition systems, providing continuous rhythm monitoring for approximately 2–4 years and enabling automated arrhythmia detection with remote transmission [55]. According to current ESC guidelines, particularly the 2018 ESC Syncope Guidelines and the 2020 ESC Atrial Fibrillation Guidelines, ILRs carry a Class I indication in patients with recurrent unexplained syncope of suspected arrhythmic origin when initial evaluation (including ECG, echocardiography, and short-term monitoring) is inconclusive [56,57]. ILRs are also recommended early in the diagnostic pathway in high-risk patients without an established diagnosis after comprehensive assessment. In addition, ILRs are indicated for long-term rhythm monitoring in patients with cryptogenic stroke to detect subclinical atrial fibrillation (Class IIa) [57]. These recommendations reflect evidence demonstrating superior diagnostic yield compared with conventional short-term monitoring, particularly for infrequent or transient arrhythmias.

Pacemaker devices combine intracardiac electrogram sensing with pacing therapy, enabling continuous atrial and ventricular rhythm diagnostics, atrial high-rate episode burden, and activity metrics. Several trials have refined pacing indication and strategies [58]. According to the 2021 ESC Guidelines on cardiac pacing and cardiac resynchronization therapy, permanent pacing carries a Class I indication in patients with symptomatic sinus node disease and in those with advanced or complete atrioventricular block, regardless of symptoms in high-grade conduction disease [59]. Pacing is also recommended in specific neuro-mediated syncope and conduction system disease scenarios, with device selection and programming tailored to underlying conduction abnormalities and patient characteristics [60,61]. Device longevity typically ranges from 7 to 12 years, depending on pacing burden.

Leadless PMK represent an evolution of conventional transvenous pacing systems, designed to eliminate leads and subcutaneous pockets, thereby reducing lead-related complications such as infection, venous obstruction, and lead fracture [62]. These devices are entirely self-contained and implanted directly within the right ventricle via a percutaneous femoral approach [63]. Current-generation systems provide single-chamber ventricular pacing and advanced sensing algorithms, with projected battery longevity typically ranging from 8 to 15 years depending on pacing burden. Leadless PMKs are particularly suitable for patients with atrial fibrillation and slow ventricular response, high infection risk, limited venous access, or prior device-related complications [64]. Although dual-chamber and atrioventricular-synchronous technologies are emerging, current indications remain more restricted compared with conventional transvenous systems, and extraction strategies in the long term remain an area of ongoing clinical investigation [65].

Conventional transvenous ICD systems incorporate multi-vector electrogram sensing optimized for ventricular arrhythmia detection. Signal processing algorithms classify rhythm morphology and rate to trigger anti-tachycardia pacing or defibrillation through intracardiac leads [66]. Battery longevity generally ranges from 5 to 10 years. These systems are strongly supported by randomized controlled trials and are a cornerstone of SCD prevention, while simultaneously functioning as high-fidelity implantable bio-signal acquisition platforms [67,68,69]. According to the 2022 ESC Guidelines on ventricular arrhythmias and the prevention of SCD and the 2023 ESC Cardiomyopathy Guidelines, ICD implantation carries a Class I indication for secondary prevention in patients with documented sustained ventricular arrhythmias and for primary prevention in patients with symptomatic HF (NYHA II–III) and LVEF ≤35% despite ≥3 months of optimal medical therapy [1,6]. In selected cardiomyopathies (e.g., *LMNA, FLNC, RBM20, PLN p.Arg14del*, desmosomal disease), ICD implantation may be considered at higher LVEF thresholds (<50%) based on genotype-specific risk stratification (Class IIa/IIb depending on risk profile). Contemporary guidelines emphasize individualized, risk-based decision-making integrating imaging, arrhythmic burden, genetics, and clinical trajectory rather than relying solely on LVEF [1,6].

In recent years, alternative lead configurations have been developed to reduce lead-related complications. The subcutaneous ICD (S-ICD) delivers defibrillation therapy via a completely extrathoracic system without transvenous leads, thereby minimizing risks of bloodstream infection and lead failure; however, it does not provide bradycardia pacing or ATP (except for limited post-shock pacing) [70,71,72]. More recently, the extravascular ICD (EV-ICD) has been introduced, featuring a substernal lead placement that enables defibrillation and anti-tachycardia pacing while avoiding transvenous intracardiac leads [73]. These novel systems expand therapeutic options, particularly in younger patients, individuals with limited venous access, or those at elevated risk of device-related infection and represent an important step toward less invasive yet functionally comprehensive defibrillation strategies [74].

CRT devices provide biventricular pacing to restore electrical synchrony in patients with HF and intraventricular conduction delay, particularly left bundle branch block [75]. CRT-P devices provide pacing alone, whereas CRT-D systems combine resynchronization therapy with defibrillation capability [76]. Device longevity generally ranges from 6 to 10 years for CRT-P and 4–8 years for CRT-D systems. According to the 2021 ESC Guidelines on cardiac pacing and cardiac resynchronization therapy, CRT carries a Class I recommendation in patients with symptomatic HF (NYHA II–IV), left ventricular ejection fraction (LVEF) ≤ 35%, and a wide QRS complex (particularly ≥150 ms) with left bundle branch block (LBBB) morphology despite optimal medical therapy. CRT should also be considered (Class IIa) in selected patients with QRS 130–149 ms and LBBB or in non-LBBB morphologies with wider QRS durations. The choice between CRT-P and CRT-D depends on the patient’s arrhythmic risk profile, underlying cardiomyopathy, life expectancy, and comorbidity burden [1,59]. In individuals with a clear indication for defibrillator therapy (e.g., primary or secondary prevention of SCD), CRT-D is generally preferred, whereas CRT-P may be appropriate in patients with lower arrhythmic risk and pacing support rather than defibrillator protection [75,76].

Hemodynamic implantable sensors represent a distinct category focused on direct pressure monitoring. The CardioMEMS HF system (Abbott, California, USA) is a permanently implantable, battery-free sensor placed in the pulmonary artery (PA) via a catheter to monitor pulmonary artery blood pressure in real-time, externally powered during data acquisition [77]. The clinical efficacy of the CardioMEMS HF pressure monitoring system has been primarily evaluated in the CHAMPION trial and the subsequent GUIDE-HF study [78,79]. Together, these trials support the role of implantable pulmonary artery pressure monitoring in reducing HF morbidity through proactive, data-driven therapy optimization. According to the 2021 ESC Guidelines for the diagnosis and treatment of acute and chronic HF, implantable pulmonary artery pressure monitoring with the CardioMEMS system has a Class IIb (Level of Evidence B) recommendation, in symptomatic HF patients, typically NYHA class III, with a previous HF hospitalization, to reduce the risk of HF hospitalizations [80].

The Cordella Pulmonary Artery (PA) Pressure Sensor (Endotronix, Inc., Illinois, USA) is another wireless, battery-free implantable hemodynamic monitoring system designed for remote management of HF. Implanted in the pulmonary artery via right heart catheterization, the sensor integrates pressure monitoring within a broader digital ecosystem and is recently FDA-approved (June 2024). The Cordella PA pressure sensor trials (SIRONA I/II, PROACTIVE-HF) demonstrate that this wireless, implantable sensor is a safe and accurate tool for monitoring HF patients [81,82]. Trials show high patient compliance, accurate readings compared to standard catheters, and a significant reduction in HF hospitalizations by guiding proactive, data-driven medication adjustments [83].

The V-LAP (vectorious left atrial pressure) system (Vectorious Medical Technologies, Tel Aviv, Israel) is also a wireless, battery-free implantable sensor designed for direct, continuous measurement of left atrial pressure (LAP) in patients with chronic HF [84]. Implanted trans-septally across the interatrial septum, the device is externally powered during data acquisition and transmits high-fidelity left atrial pressure (LAP) waveforms to a remote monitoring platform, aiming to enable earlier detection of congestion and more individualized, pressure-guided therapy optimization [85,86]. Early feasibility data from the multicenter VECTOR-HF first-in-human study demonstrated high implantation success, reliable waveform acquisition with good correlation to invasive measurements, and an acceptable safety profile [87]. The system has obtained CE marking in Europe, and it remains investigational in the United States.

### 4.3. Contact-Free and Ambient Sensors of Cardiac Monitoring

Contact-free and ambient cardiac monitoring systems represent a rapidly evolving paradigm in unobtrusive cardiovascular surveillance, enabling passive, continuous assessment without direct skin contact, wearable adherence, or invasive implantation. These technologies rely on diverse physical principles to capture subtle physiological signals associated with cardiac activity.

Mechanical recoil–based systems such as ballistocardiography (BCG), embedded in beds, chairs, or even smart toilets, detect micro-forces generated by blood ejection into the great vessels and can derive HR, HRV, and surrogate stroke volume metrics [88,89,90]. Seismocardiography (SCG) and related vibration-based techniques measure chest wall micro-accelerations linked to mechanical cardiac events, allowing estimation of systolic time intervals and potentially contractility-related indices [91,92,93].

Electromagnetic sensing approaches—including Doppler radar, ultra-wideband radar, and millimeter-wave (mmWave) systems—analyze thoracic displacement via reflected radiofrequency signals, enabling contactless HR and respiratory monitoring even through clothing or bedding [94,95,96,97]. Optical strategies such as remote photoplethysmography (rPPG) extract pulsatile blood flow information from subtle skin color fluctuations captured by standard RGB cameras, while infrared thermal imaging and LiDAR-based depth sensing quantify perfusion-driven temperature oscillations or micro-thoracic movements [98,99,100]. Additional experimental modalities include contact-free acoustic cardiography, capacitive ECG through clothing, environmental RF/WiFi signal perturbation analysis, floor vibration sensors, and laser speckle imaging of microvascular flow [101]. Increasingly, multimodal AI-driven platforms integrate camera, radar, and mechanical sensors to enhance signal fidelity and derive composite digital biomarkers [102].

Importantly, these systems hold substantial potential for in-hospital deployment, including general wards and intensive care units (ICUs), where continuous, non-contact monitoring could reduce wiring burden, improve patient comfort, and enable early detection of clinical deterioration. In high-acuity settings, ambient sensors may complement conventional telemetry by providing redundancy, reducing alarm fatigue through AI-assisted filtering, and facilitating surveillance of multiple patients simultaneously. As such, contact-free monitoring may contribute to scalable, low-friction cardiovascular surveillance models within modern hospital infrastructures [103]. Table 4 shows an overview of currently available contact-free and ambient sensors for cardiac monitoring cardiac devices.

Although promising, contact-free systems currently lack robust validation for ventricular arrhythmia detection in high-risk genetic cardiomyopathies. Their principal role may initially lie in complementary hemodynamic and autonomic trend monitoring rather than primary arrhythmic diagnostics.

## 5. AI and Signal Processing in Remote Monitoring

The rapid expansion of sensor-based cardiac monitoring has fundamentally altered the scale and complexity of physiological data generated in routine care. Continuous electrocardiographic, hemodynamic, and behavioral data streams offer unprecedented opportunities for arrhythmic surveillance, but they also pose substantial analytical challenges. In this context, AI and advanced signal processing techniques have emerged as essential components of modern remote monitoring systems, enabling the transformation of high-volume, high-noise sensor data into clinically actionable information [104].

One of the earliest and most mature applications of AI in remote cardiac monitoring is automated arrhythmia detection. Machine learning and deep learning algorithms have been developed to classify cardiac rhythms, identify atrial and ventricular arrhythmias, and reduce false-positive alerts generated by traditional rule-based systems. These approaches are particularly valuable in long-term monitoring, where even low false-positive rates can translate into substantial clinical workload. In implantable cardiac monitors and CIED-based remote monitoring platforms, AI-enhanced algorithms have demonstrated the ability to preserve arrhythmia detection sensitivity while significantly reducing the number of electrograms requiring manual review [105,106]. This balance between sensitivity and efficiency is critical for sustainable implementation of intensive monitoring strategies, especially in populations requiring lifelong surveillance.

A major contributor to false-positive alerts in long-term monitoring systems is signal quality degradation during ambulatory recording. Motion artifacts, poor electrode–skin contact, baseline wander, and electromagnetic interference can introduce noise or distort morphological features of the ECG signal. In wearable photoplethysmography systems, motion-induced optical artifacts and variations in peripheral perfusion may similarly lead to erroneous rhythm classification [107,108]. Signal quality indices and artifact detection algorithms are therefore increasingly integrated into AI-enabled monitoring pipelines to automatically assess recording reliability before arrhythmia classification is performed.

Similarly, AI-driven ECG analysis has been applied in wearable and ambulatory monitoring devices to enable real-time or near-real-time arrhythmia pre-diagnosis [109]. These systems leverage convolutional neural networks and other pattern-recognition architectures to classify rhythm disturbances directly from raw or minimally processed ECG signals, facilitating early identification of clinically relevant arrhythmias in remote settings [110].

In real-world remote monitoring environments, ECG recordings frequently contain noise and artifacts that can affect automated arrhythmia detection. Consequently, most AI-based monitoring pipelines incorporate signal preprocessing steps prior to classification. These steps may include baseline wander removal, band-pass filtering to suppress high-frequency noise, normalization of signal amplitude, and artifact detection algorithms that exclude low-quality segments. Signal quality assessment is particularly important in wearable systems where patient movement, electrode displacement, and environmental noise can degrade ECG morphology. Robust preprocessing therefore represents a critical component for maintaining reliable AI-based rhythm classification in ambulatory monitoring settings [111,112].

Beyond event detection, a key strength of AI lies in its capacity to identify complex temporal patterns across longitudinal data streams. In contrast to conventional monitoring approaches that focus on isolated arrhythmic episodes, AI-based models can analyze trends in ectopic burden, HRV, conduction intervals, activity levels, and hemodynamic parameters over time. This capability aligns closely with the pathophysiology of high-risk cardiomyopathy genotypes, in which arrhythmic risk often evolves gradually rather than manifesting abruptly. While these systems have primarily been developed in HF populations, their underlying principle—that adverse cardiovascular events are preceded by detectable, multidimensional changes—is highly relevant to arrhythmic risk surveillance in inherited cardiomyopathies [113,114,115].

In genotype-defined cardiomyopathy populations, AI-based analytical frameworks may provide additional value beyond conventional arrhythmia detection. By integrating longitudinal rhythm data with genotype-specific clinical features, AI models may help identify early trajectories of electrical instability characteristic of particular genetic substrates. For instance, progressive PR prolongation and conduction disease in *LMNA* carriers, increasing ventricular ectopy in *FLNC* truncating variants, or activity-triggered ventricular arrhythmias in desmosomal disease may represent distinct digital phenotypes. AI-driven pattern recognition across multimodal datasets—including ECG morphology, ectopic burden trends, heart-rate variability, and activity context—may therefore support genotype-informed risk stratification and improve timing of prophylactic interventions such as implantable cardioverter-defibrillator implantation [116,117].

Deep learning algorithms have demonstrated high accuracy in detecting atrial fibrillation and ventricular arrhythmias from single-lead ECG and PPG signals [99]. In the context of inherited cardiomyopathies, AI offers the potential to identify subtle, genotype-specific electrical signatures, such as conduction slowing, repolarization abnormalities, or changes in ectopic burden over time.

As remote monitoring ecosystems increasingly incorporate multiple sensor modalities, AI plays a critical role in data integration and prioritization. Internet of Medical Things (IoMT) frameworks and cloud-based analytics platforms have been proposed to aggregate data from wearable, implantable, and ambient sensors, facilitating centralized analysis and longitudinal risk assessment [118]. Within these architectures, AI algorithms can assign dynamic risk scores, filter clinically irrelevant data, and escalate alerts based on individualized thresholds [118]. This approach is particularly important in high-risk cardiomyopathy populations, where continuous monitoring may be justified but unsustainable without intelligent data reduction and triage mechanisms.

### Limitations and Ethical Considerations

Despite promising results in controlled datasets, several challenges remain for the clinical deployment of AI-based arrhythmia detection models. Many algorithms are trained on datasets derived from single device platforms or limited patient populations, which may reduce generalizability across different clinical contexts. External validation using independent multicenter cohorts is therefore essential to assess real-world performance. Furthermore, deep learning models are susceptible to overfitting when trained on small or imbalanced datasets, particularly in rare disease populations such as inherited cardiomyopathies [119,120]. Although AI-driven triage and prioritization have demonstrated the potential to reduce manual review workload while preserving arrhythmia detection sensitivity, these systems are not infallible [121]. Each monitoring modality presents distinct advantages and limitations. Wearable sensors offer non-invasive continuous monitoring but are susceptible to motion artifacts and variable patient adherence. Implantable devices provide highly reliable long-term rhythm detection but involve procedural risks and higher costs. Ambient and contact-free monitoring systems remain promising but currently face limitations in signal fidelity and validation across diverse clinical settings. False-positive arrhythmia alerts may lead to unnecessary anxiety and downstream testing, emphasizing the importance of explainability and clinician oversight. These trade-offs highlight the need for individualized monitoring strategies tailored to patient risk profile and disease stage [122].

Most AI models for arrhythmia detection are trained on large heterogeneous cardiovascular populations, whereas high-risk genotypes such as *LMNA, RBM20*, or *PLN p.Arg14del* represent rare subsets with distinct electrophysiological signatures. Model generalizability may therefore be compromised, particularly in detecting subtle genotype-specific conduction slowing or repolarization abnormalities. Furthermore, continuous-learning systems may be exposed to model drift over time, especially when deployed in longitudinal remote monitoring environments. Explainable AI frameworks are particularly relevant in this context, as clinical decisions such as prophylactic ICD implantation require transparency and physician interpretability rather than black-box outputs.

Several strategies may help address these limitations in future research. First, the development of genotype-specific datasets and international registries could enable the training of AI models tailored to rare cardiomyopathy populations. Collaborative initiatives integrating genetic registries with remote monitoring data may facilitate the creation of large, harmonized datasets suitable for model development and validation. Second, hybrid modeling approaches combining mechanistic clinical variables—such as genotype, fibrosis burden on cardiac magnetic resonance, and family history—with longitudinal sensor-derived digital biomarkers may improve predictive performance. Third, federated learning frameworks may allow AI models to be trained across multiple institutions without centralized data sharing, thereby improving generalizability while preserving data privacy. These strategies may help overcome the challenges of limited sample size and heterogeneity inherent to inherited cardiomyopathy research.

The deployment of continuous AI-enabled monitoring raises important regulatory and ethical questions, particularly in the context of lifelong surveillance for inherited diseases [123,124]. AI algorithms used for arrhythmia detection or risk prediction are increasingly classified as software as a medical device (SaMD), subject to evolving oversight frameworks that require transparency, performance validation, and post-market surveillance [125]. Ensuring data security, patient privacy, and compliance with evolving regulatory frameworks is therefore a fundamental requirement. To mitigate these concerns, the integration of AI-enabled monitoring should be guided by clear clinical governance structures, transparent communication strategies, and equitable access policies, ensuring that technological innovation translates into meaningful and responsible patient benefit.

## 6. Conclusions

In conclusion, this narrative review highlights that AI-enabled sensor technologies are reshaping the paradigm of arrhythmic surveillance in patients carrying high-risk cardiomyopathy genotypes, in whom malignant ventricular arrhythmias and SCD often occur independently of overt structural deterioration. By integrating genotype-driven risk stratification with continuous physiological monitoring, wearable, implantable, and contact-free sensors provide access to dynamic electrical and hemodynamic biomarkers that cannot be captured through conventional intermittent assessments. Multisensor implantable platforms, pulmonary artery pressure monitoring systems, AI-enhanced implantable loop recorders, and consumer-grade wearables collectively demonstrate the feasibility of early detection of electrical instability, subclinical decompensation, and arrhythmic events, while reducing clinical workload through automated data triage. However, most current evidence derives from heterogeneous HF or general arrhythmia populations, with limited prospective validation in genetically defined cohorts such as *LMNA, FLNC, RBM20, PLN*, or desmosomal variant carriers. Algorithm generalizability, signal quality in ambulatory settings, data overload, regulatory constraints, and ethical considerations related to continuous surveillance remain significant challenges. AI-enabled sensor ecosystems should not be viewed merely as extensions of traditional rhythm monitoring, but as tools for dynamic risk recalibration over time.

Future research should therefore prioritize the development of integrated monitoring ecosystems combining genetic information, multimodal sensor data, and AI-based analytics, supported by large collaborative datasets and rigorous clinical validation. Such approaches may enable the identification of genotype-specific digital biomarkers and facilitate personalized arrhythmic risk stratification in inherited cardiomyopathy populations.

## Figures and Tables

**Table 1:** Genotype-Specific Arrhythmic Phenotype and Monitoring Priorities.

Genotype	Arrhythmic Pattern	Structural Hallmarks	Monitoring Priority	Suggested Early Sensor Strategy
*LMNA*	Conduction disease, NSVT, early AVB [16]	Mid-wall fibrosis, Mild LV dilation [17,18]	Early electrical instability detection	Wearable sensors (smartwatch, ECG patch) → early ILR if syncope or NSVT detected
*FLNC* (truncating variants)	Ventricular arrhythmias disproportionate to LVEF [19]	Extensive myocardial fibrosis [20,21]	NSVT detection and burden tracking	Extended ECG patch → ILR if NSVT detected
*RBM20*	Early malignant ventricular arrhythmias [23,35]	Aggressive dilated cardiomyopathy [24,26]	Pre-phenotypic rhythm monitoring	Wearable sensors, Long-term ambulatory ECG
*PLN p.Arg14del*	Low-voltage ECG, ventricular arrhythmias [36]	Mixed DCM/arrhythmogenic phenotype [37]	Conduction and ectopy progression monitoring	Serial ECG patch + HRV longitudinal trends
Desmosomal genes (*DSP, PKP2, DSG2, DSC2, TMEM43*)	Exercise-triggered VT/VF [31,38]	RV/LV fibrofatty replacement [39]	Activity-linked arrhythmia detection	Wearable ECG, ILR in high-risk cases (risk stratification)

Abbreviations: VT, ventricular tachycardia; VF, ventricular fibrillation; NSVT, non-sustained ventricular tachycardia; AVB, atrio-ventricular block; DCM, dilated cardiomyopathy; LV, left ventricle; RV, right ventricle; LVEF, LV ejection fraction; HRV, heart rate variability; ILR, implantable loop recorder.

**Table 2:** Wearable Sensors for Cardiac Monitoring and Regulatory Status.

Category	Sensor Technology	Measured Signals	Clinical Applications	Representative Examples
ECG Patch (Single-Lead)	Adhesive dry-electrode ECG	Up to 14 days of continuous ECG	Arrhythmia diagnosis	Zio Patch® 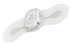
ECG Patch (P-wave-centric)	Adhesive single-electrode ECG	Up to 14 days of continuous ECG (2–12 lead)	Designed for accurate P-wave recordings, for AF detection	Bardy CAM® 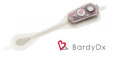
Extended ECG Wearable Monitor	Adhesive single-electrode ECG	7–30 days single lead ECG	Paroxysmal AF detection	Preventice BodyGuardian® 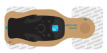
Chest Strap ECG	ECG electrodes + accelerometer	HR, HRV	Exercise monitoring	Polar H10® 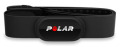
Smartwatch (ECG-enabled)	Single lead ECG + PPG	Single-lead ECG, HR	AF screening	Apple Watch 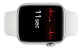
Smart Ring	PPG + temperature + accelerometer	HR, HRV, sleep	Autonomic and sleep tracking	Oura Ring® 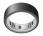
Smart Garment (Textile ECG)	Embedded textile electrodes	ECG, respiration	Long-term ECG monitoring	Hexoskin® 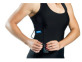
Wearable BP Monitor (Cuff-based)	Oscillometric wrist cuff	Blood pressure	Hypertension monitoring	Omron® HeartGuide 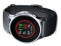
Bioimpedance Wearable	Thoracic impedance	Fluid status	HF decompensation	Research platforms

Abbreviations: AF, atrial fibrillation; HF, heart failure; HR, heart rate; HRV, heart rate variability; PPG, photoplethysmography. Devices manufacturers (city, country): Zio patch, iRhythm Technologies, Inc. (San Francisco, California); Bardy Cam, Bardy Diagnostics (Seattle, Washington, USA); Preventice Body Guardian, Boston Scientific (Massachusetts, USA); Polar H10, Polar Electro Oy (Kempele, Finland); Apple Watch, Apple Inc. (Cupertino, California, USA); Oura Ring, Oura Health Oy, (Oulu, Finland); Hexoskin, (Montreal, Quebec); Omron Hear Guide, OMRON Healthcare Co., Ltd. (Kyoto, Japan).

**Table 3:** Implantable Cardiac Monitoring and Therapeutic Devices.

Device Category	Primary Function	Longevity	Clinical Indications	Representative Examples	FDA Approval
ILR	Continuous subcutaneous single lead rhythm diagnostics	2–4 years	Unexplained syncope, cryptogenic stroke, AF detection	Reveal LINQ®, BioMonitor® 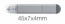	Yes
TV-PMK	Atrial/ventricular pacing, rhythm diagnostics	7–12 years	Bradycardia, Sinus node disease, AVB	Azure®, Assurity® 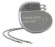	Yes
Leadless PMK	Single-chamber bradycardia therapy (intracardiac)	8–15 years	Reduces lead-related complications (infection, venous obstruction) and lead fracture	Micra®, Aveir® 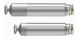	Yes
TV-ICD	Defibrillation, anti-tachycardia pacing, rhythm diagnostics	5–10 years	Primary/secondary SCD prevention	Visia AF®, Dynagen® 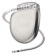	Yes
EV-ICD	Substernal lead defibrillation, pacing capability, rhythm diagnostics	6–10 years	Primary/secondary SCD prevention with no intracardiac leads	Aurora® 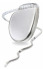	Yes
S-ICD	Subcutaneous defibrillation, no chronic pacing, rhythm diagnostics	7–9 years	SCD prevention when pacing not required	EMBLEM® 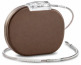	Yes
CRT-P	Biventricular pacing, rhythm diagnostics	6–10 years	HFrEF with conduction delay	Viva® 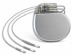	Yes
CRT-D	Biventricular pacing, ICD therapy, rhythm diagnostics	4–8 years	HFrEF with arrhythmic risk	Claria® 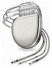	Yes
V-LAP	Left atrial pressure monitoring	Battery-free, wirelessly powered	Advanced HF monitoring	V-LAP System 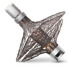	Investigational (US)
Cardio-MEMS HF System	PA pressure monitoring	Battery-free, wireless powered	HF hospitalization reduction	CardioMEMS® HF System 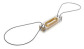	Yes
Cordella PA Sensor	PA pressure monitoring	Battery-free, wireless powered	HF management	Cordella® System 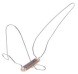	Yes

Abbreviations: ILR, Implantable Loop Recorder; PMK, pacemaker; TV-PMK, transvenous PMK; AVB, atrio-ventricular block; ICD, implantable cardiac defibrillator; SCD, sudden cardiac death; TV-ICD, transvenous ICD; S-ICD, subcutaneous ICD; EV-ICD, extravascular ICD; CRT, cardiac resynchronization therapy pacemaker; CRT-D, cardiac resynchronization therapy defibrillator; V-LAP, Vectorious left atrial pressure; PA, pulmonary pressure; AF, atrial fibrillation; HF, heart failure; HFrEF, HF with reduced ejection fraction (≤35%). Manufacturers (city, country): Reveal LNQ, Visia AF, Micra, Azure, Aurora, Viva, Claria: Medtronic (Dublin, Ireland); Biomonitor, Biotronik (Berlin, Germany); Assurity, Aveir, CardioMEMS: Abbott (California, USA); Dynagen, EMBLEM: Boston Scientific Corporation (Massachusetts, USA); V-LAP System, Vectorious Medical Technologies (Tel Aviv, Israel); Cordella System: Endotronix, Inc. (Illinois, USA).

**Table 4:** Contact-Free and Ambient Sensors for Cardiac Monitoring.

Sensor Category	Physical Principle	Measured Signals	Cardiac Parameters Extracted	Clinical Applications	Maturity Level
BCG Bed-based	Micro-force mattress sensors	Body recoil from cardiac ejection	HR, HRV, stroke volume surrogates	HF monitoring, sleep cardiology	Clinical research/limited commercial
SCG Radar-enhanced	Chest wall vibration detection	Mechanical cardiac timing	Systolic time intervals	HF decompensation prediction	Research
Doppler Radar	Microwave Doppler shift	Chest wall displacement	HR, respiration rate	Remote ICU, sleep labs	Research → early clinical
Camera-based PPG	Optical reflectance via RGB camera	Skin color micro-variations	HR, HRV	Telemedicine, AF screening	Early clinical validation
Infrared Thermal Imaging	Temperature variation mapping	Facial perfusion dynamics	HR estimation	Remote triage	Research
LiDAR-based Cardiography	Optical depth sensing	Micro thoracic displacement	HR	Experimental remote monitoring	Research
Smart Bed Systems	Integrated BCG + pressure mapping	Cardiorespiratory signals	HR, HRV	Hospital ward monitoring	Commercial (limited validation)
Capacitive ECG (cECG)	Electric field sensing through clothing	ECG waveform	Rhythm detection	Driver monitoring, niche clinical	Research/niche clinical
Ultra-wideband Radar	RF impulse reflection	Motion detection	HR	ICU and sleep monitoring	Research
AI-Fusion Ambient Systems	Multimodal (camera + radar + BCG)	Combined cardiorespiratory signals	HR, HRV, arrhythmia suspicion	Smart hospital rooms	Emerging

Abbreviations: HR, heart rate; HRV, heart rate variability; BCG, ballistocardiography; SCG, Seismocardiography; PPG, photoplethysmography; ICU, intensive care unit.

## Data Availability

No new data were created or analyzed in this study.

## References

[1] Arbelo E., Protonotarios A., Gimeno J.R., Arbustini E., Barriales-Villa R., Basso C., Bezzina C.R., Biagini E., Blom N.A., de Boer R.A. (2023). 2023 ESC Guidelines for the Management of Cardiomyopathies. Eur. Heart J..

[2] Elliott P., Schunkert H., Bondue A., Behr E., Carrier L., Van Duijn C., García-Pavía P., van der Harst P., Kavousi M., Loeys B. (2025). Integration of Genetic Testing into Diagnostic Pathways for Cardiomyopathies: A Clinical Consensus Statement by the ESC Council on Cardiovascular Genomics. Eur. Heart J..

[3] Sterner R.M., Coon L.M., Black J.L., Moyer A.M., Maleszewski J.J., Baudhuin L.M. (2026). Autosomal-Recessive LMNA Dilated Cardiomyopathy. JACC Case Rep..

[4] Charron P., Elliott P.M., Gimeno J.R., Caforio A.L.P., Kaski J.P., Tavazzi L., Tendera M., Maupain C., Laroche C., Rubis P. (2018). The Cardiomyopathy Registry of the EURObservational Research Programme of the European Society of Cardiology: Baseline Data and Contemporary Management of Adult Patients with Cardiomyopathies. Eur. Heart J..

[5] Smith E.D., Lakdawala N.K., Papoutsidakis N., Aubert G., Mazzanti A., McCanta A.C., Agarwal P.P., Arscott P., Dellefave-Castillo L.M., Vorovich E.E. (2020). Desmoplakin Cardiomyopathy, a Fibrotic and Inflammatory Form of Cardiomyopathy Distinct from Typical Dilated or Arrhythmogenic Right Ventricular Cardiomyopathy. Circulation.

[6] Zeppenfeld K., Tfelt-Hansen J., De Riva M., Winkel B.G., Behr E.R., Blom N.A., Charron P., Corrado D., Dagres N., De Chillou C. (2022). 2022 ESC Guidelines for the Management of Patients with Ventricular Arrhythmias and the Prevention of Sudden Cardiac Death. Eur. Heart J..

[7] Gaoudam N., Sakhamudi S.K., Kamal B., Addla N., Reddy E.P., Ambala M., Lavanya K., Palaparthi E.C., Bhattam A., Periasamy P. (2026). Wearable Devices and AI-Driven Remote Monitoring in Cardiovascular Medicine: A Narrative Review. Cureus.

[8] Banerjee A. (2025). Artificial Intelligence Enabled Mobile Health Technologies in Arrhythmias-an Opinion Article on Recent Findings. Front. Cardiovasc. Med..

[9] Xie H., Yang L., Jiang B., Huang Z., Lin Y. (2025). State-of-the-Art Wearable Sensors for Cardiovascular Health: A Review. npj Cardiovasc. Health.

[10] Ciarambino T., Menna G., Sansone G., Giordano M. (2021). Cardiomyopathies: An Overview. Int. J. Mol. Sci..

[11] Sorella A., Galanti K., Iezzi L., Gallina S., Mohammed S.F., Sekhri N., Akhtar M.M., Prasad S.K., Chahal C.A.A., Ricci F. (2025). Diagnosis and Management of Dilated Cardiomyopathy: A Systematic Review of Clinical Practice Guidelines and Recommendations. Eur. Heart J. Qual. Care Clin. Outcomes.

[12] Iezzi L., Sorella A., Galanti K., Gallina S., Chahal A.A., Bauce B., Cipriani A., Providencia R., Lopes L.R., Ricci F. (2025). Arrhythmogenic Cardiomyopathy Diagnosis and Management: A Systematic Review of Clinical Practice Guidelines and Recommendations with Insights for Future Research. Eur. Heart J. Qual. Care Clin. Outcomes.

[13] Cardoso I., Nunes S., Brás P., Viegas J.M., Marques Antunes M., Ferreira A., Almeida I., Custódio I., Trigo C., Laranjo S. (2025). The Contribution of Genetics to the Understanding and Management of Cardiomyopathies: Part 1. Rev. Port. Cardiol..

[14] Kontorovich A.R. (2023). Approaches to Genetic Screening in Cardiomyopathies. JACC Heart Fail..

[15] Parikh V.N., Day S.M., Lakdawala N.K., Adler E.D., Olivotto I., Seidman C.E., Ho C.Y. (2025). Advances in the Study and Treatment of Genetic Cardiomyopathies. Cell.

[16] Lazarte J., Jurgens S.J., Choi S.H., Khurshid S., Morrill V.N., Weng L.C., Nauffal V., Pirruccello J.P., Halford J.L., Hegele R.A. (2022). LMNA Variants and Risk of Adult-Onset Cardiac Disease. J. Am. Coll. Cardiol..

[17] Balakrishnan I.D., Lakdawala N.K. (2025). Contemporary Insights into LMNA Cardiomyopathy. Curr. Cardiol. Rep..

[18] Rosario K.F., Karra R., Amos K., Landstrom A.P., Lakdawala N.K., Brezitski K., Kim H., Devore A.D. (2023). LMNA Cardiomyopathy: Important Considerations for the Heart Failure Clinician. J. Card. Fail..

[19] Carruth E.D., Qureshi M., Alsaid A., Kelly M.A., Calkins H., Murray B., Tichnell C., Sturm A.C., Baras A., Lester Kirchner H. (2022). Loss-of-Function FLNC Variants Are Associated with Arrhythmogenic Cardiomyopathy Phenotypes When Identified Through Exome Sequencing of a General Clinical Population. Circ. Genom. Precis. Med..

[20] Ader F., De Groote P., Réant P., Rooryck-Thambo C., Dupin-Deguine D., Rambaud C., Khraiche D., Perret C., Pruny J.F., Mathieu-Dramard M. (2019). FLNC Pathogenic Variants in Patients with Cardiomyopathies: Prevalence and Genotype-Phenotype Correlations. Clin. Genet..

[21] Verdonschot J.A.J., Vanhoutte E.K., Claes G.R.F., Helderman-van den Enden A.T.J.M., Hoeijmakers J.G.J., Hellebrekers D.M.E.I., de Haan A., Christiaans I., Lekanne Deprez R.H., Boen H.M. (2020). A Mutation Update for the FLNC Gene in Myopathies and Cardiomyopathies. Hum. Mutat..

[22] Ortiz-Genga M.F., Cuenca S., Dal Ferro M., Zorio E., Salgado-Aranda R., Climent V., Padrón-Barthe L., Duro-Aguado I., Jiménez-Jáimez J., Hidalgo-Olivares V.M. (2016). Truncating FLNC Mutations Are Associated With High-Risk Dilated and Arrhythmogenic Cardiomyopathies. J. Am. Coll. Cardiol..

[23] Van Den Hoogenhof M.M.G., Beqqali A., Amin A.S., Van Der Made I., Aufiero S., Khan M.A.F., Schumacher C.A., Jansweijer J.A., Van Spaendonck-Zwarts K.Y., Remme C.A. (2018). RBM20 Mutations Induce an Arrhythmogenic Dilated Cardiomyopathy Related to Disturbed Calcium Handling. Circulation.

[24] Hermida A., Ader F., Millat G., Jedraszak G., Vogel L., Garçon L., Maury P., Fay F., Beyls C., Bréhin A.C. (2025). RBM20 Gene in Patients With Cardiomyopathy: Phenotypic Expression for Loss-of-Function Versus Hotspot Variants. Circ. Heart Fail..

[25] Gregorich Z.R., Zhang Y., Kamp T.J., Granzier H.L., Guo W. (2024). Mechanisms of RBM20 Cardiomyopathy: Insights from Model Systems. Circ. Genom. Precis. Med..

[26] Cannie D.E., Protonotarios A., Bakalakos A., Syrris P., Lorenzini M., De Stavola B., Bjerregaard L., Dybro A.M., Hey T.M., Hansen F.G. (2023). Risks of Ventricular Arrhythmia and Heart Failure in Carriers of RBM20 Variants. Circ. Genom. Precis. Med..

[27] van Drie E., Jongbloed J.D.H., Hoorntje E., van der Zwaag P.A., Cox M.G.P.J., Deprez R.H.L., Houweling A.C., Proost V.P., Wilde A.A.M., Dooijes D. (2025). Additional Genetic Variants in Cardiomyopathy Patients with the Pathogenic PLN p.(Arg14del) Founder Variant. Int. J. Cardiol..

[28] Stege N.M., de Boer R.A., Makarewich C.A., van der Meer P., Silljé H.H.W. (2024). Reassessing the Mechanisms of PLN-R14del Cardiomyopathy: From Calcium Dysregulation to S/ER Malformation. JACC Basic Transl. Sci..

[29] de Brouwer R., te Rijdt W.P., Hoorntje E.T., Amin A., Asselbergs F.W., Cox M.G.P.J., van der Heijden J.F., Hillege H., Karper J.C., Mahmoud B. (2023). A Randomized Controlled Trial of Eplerenone in Asymptomatic Phospholamban p.Arg14del Carriers. Eur. Heart J..

[30] Nagyova E., Hoorntje E.T., te Rijdt W.P., Bosman L.P., Syrris P., Protonotarios A., Elliott P.M., Tsatsopoulou A., Mestroni L., Taylor M.R.G. (2023). A Systematic Analysis of the Clinical Outcome Associated with Multiple Reclassified Desmosomal Gene Variants in Arrhythmogenic Right Ventricular Cardiomyopathy Patients. J. Cardiovasc. Transl. Res..

[31] James C.A., Bhonsale A., Tichnell C., Murray B., Russell S.D., Tandri H., Tedford R.J., Judge D.P., Calkins H. (2013). Exercise Increases Age-Related Penetrance and Arrhythmic Risk in Arrhythmogenic Right Ventricular Dysplasia/Cardiomyopathy-Associated Desmosomal Mutation Carriers. J. Am. Coll. Cardiol..

[32] Fressart V., Duthoit G., Donal E., Probst V., Deharo J.C., Chevalier P., Klug D., Dubourg O., Delacretaz E., Cosnay P. (2010). Desmosomal Gene Analysis in Arrhythmogenic Right Ventricular Dysplasia/Cardiomyopathy: Spectrum of Mutations and Clinical Impact in Practice. Europace.

[33] Cardoso I., Melo M., Brás P., Viegas J.M., Almeida I., Nunes S., Custódio I., Trigo C., Laranjo S., Graça R. (2025). The Contribution of Genetics to the Understanding and Management of Cardiomyopathies: Part 2. Rev. Port. De Cardiol..

[34] Wieczorek D.F. (2024). Dilated Cardiomyopathy—Exploring the Underlying Causes. Med. Res. Arch..

[35] Garmany R., Castrichini M., Neves R., Pereira N.L., MacIntyre C., Schneider J.W., Ackerman M.J., Giudicessi J.R. (2025). Age at Onset and Clinical Course of RBM20-Mediated Cardiomyopathy. Sci. Rep..

[36] de Brouwer R., Meems L.M.G., Verstraelen T.E., Mahmoud B., Proost V., Wilde A.A.M., Bosman L.P., van Drie E., van der Zwaag P.A., van Tintelen J.P. (2022). Sex-Specific Aspects of Phospholamban Cardiomyopathy: The Importance and Prognostic Value of Low-Voltage Electrocardiograms. Heart Rhythm.

[37] Jiang X., Xu Y., Sun J., Wang L., Guo X., Chen Y. (2020). The Phenotypic Characteristic Observed by Cardiac Magnetic Resonance in a PLN-R14del Family. Sci. Rep..

[38] Prior D., La Gerche A. (2020). Exercise and Arrhythmogenic Right Ventricular Cardiomyopathy. Heart Lung Circ..

[39] Corrado D., Migliore F., Zorzi A. (2021). Arrhythmic Risk Stratification in Arrhythmogenic Cardiomyopathy: New Predictors for Left-Sided Variants?. Eur. Heart J..

[40] Bluemke D.A., James C.A., te Riele A.S.J.M. (2020). Risk Stratification in Arrhythmogenic Right Ventricular Cardiomyopathy: Not a One-Size-Fits-All Strategy. J. Am. Coll. Cardiol..

[41] Crea F. (2025). Genetic Testing in Cardiomyopathies and New Therapeutic Target in Cardiac Remodelling. Eur. Heart J..

[42] Sridhar A.R., Cheung J.W., Lampert R., Silva J.N.A., Gopinathannair R., Sotomonte J.C., Tarakji K., Fellman M., Chrispin J., Varma N. (2024). State of the Art of Mobile Health Technologies Use in Clinical Arrhythmia Care. Commun. Med..

[43] Bisignani G., Cheung J.W., Rordorf R., Kutyifa V., Hofer D., Berti D., Di Biase L., Martens E., Russo V., Vitillo P. (2024). Implantable Cardiac Monitors: Artificial Intelligence and Signal Processing Reduce Remote ECG Review Workload and Preserve Arrhythmia Detection Sensitivity. Front. Cardiovasc. Med..

[44] Chiu C.S.L., Gerrits W., Guglielmo M., Cramer M.J., van der Harst P., van Es R., Meine M. (2025). From Clinic to Cloud: Efficacy of AI-Assisted Remote Monitoring of Patients With Implantable Cardiac Devices. Pacing Clin. Electrophysiol..

[45] Skjølsvik E.T., Hasselberg N.E., Dejgaard L.A., Lie Ø.H., Andersen K., Holm T., Edvardsen T., Haugaa K.H. (2020). Exercise Is Associated With Impaired Left Ventricular Systolic Function in Patients With Lamin A/C Genotype. J. Am. Heart Assoc..

[46] Pedroso A.F., Khera R. (2025). Leveraging AI-Enhanced Digital Health with Consumer Devices for Scalable Cardiovascular Screening, Prediction, and Monitoring. npj Cardiovasc. Health.

[47] Alugubelli N., Abuissa H., Roka A. (2022). Wearable Devices for Remote Monitoring of Heart Rate and Heart Rate Variability—What We Know and What Is Coming. Sensors.

[48] Elgendi M., Jost E., Alian A., Fletcher R.R., Bomberg H., Eichenberger U., Menon C. (2024). Photoplethysmography Features Correlated with Blood Pressure Changes. Diagnostics.

[49] Hama F.M., Rocha E.G., Azeka E. (2025). Genetic Cardiomyopathies: An Overview of the Main Associated Pathogenic Mutations. ABC Heart Fail Cardiomyop.

[50] Hughes A., Shandhi M.M.H., Master H., Dunn J., Brittain E. (2023). Wearable Devices in Cardiovascular Medicine. Circ. Res..

[51] Ren H., Jing F., Ma Y., Wang R., He C., Wang Y., Zhou J., Sun Y. (2025). Harnessing Artificial Intelligence of Things for Cardiac Sensing: Current Advances and Network-Based Perspectives. Front. Public Health.

[52] Jin Y., Che W., Yang J., Chang S., Bao W., Ren X., Yu P., Hou A. (2025). Classification, Diagnosis, and Prognosis of Cardiomyopathy: A Comprehensive Narrative Review. Rev. Cardiovasc. Med..

[53] Rao A., Bennett S. (2022). Cardiac Implantable Electronic Devices: An Overview for Primary Care. Br. J. Gen. Pract..

[54] Al-Khatib S.M. (2024). Cardiac Implantable Electronic Devices. N. Engl. J. Med..

[55] Bisignani A., De Bonis S., Mancuso L., Ceravolo G., Bisignani G. (2019). Implantable Loop Recorder in Clinical Practice. J. Arrhythm..

[56] Brignole M., Moya A., De Lange F.J., Deharo J.C., Elliott P.M., Fanciulli A., Fedorowski A., Furlan R., Kenny R.A., Martín A. (2018). 2018 ESC Guidelines for the Diagnosis and Management of Syncope. Eur. Heart J..

[57] Hindricks G., Potpara T., Kirchhof P., Kühne M., Ahlsson A., Balsam P., Bauersachs J., Benussi S., Brandes A., Braunschweig F. (2021). 2020 ESC Guidelines for the Diagnosis and Management of Atrial Fibrillation Developed in Collaboration with the European Association for Cardio-Thoracic Surgery (EACTS). Eur. Heart J..

[58] DeForge W.F. (2019). Cardiac Pacemakers: A Basic Review of the History and Current Technology. J. Vet. Cardiol..

[59] Glikson M., Nielsen J.C., Leclercq C., Kronborg M.B., Michowitz Y., Auricchio A., Barbash I.M., Barrabés J.A., Boriani G., Braunschweig F. (2021). 2021 ESC Guidelines on Cardiac Pacing and Cardiac Resynchronization Therapy. Eur. Heart J..

[60] Sutton R., Prakash A. (2025). Physiological Pacing: Historical Review With an Eye to the Future. J. Cardiovasc. Electrophysiol..

[61] Wu Y., Xu H., Tu X., Gao Z. (2023). Review of the Epidemiology, Pathogenesis and Prevention of Atrial Fibrillation after Pacemaker Implantation. Adv. Clin. Exp. Med..

[62] El-Chami M.F., Cunnane R. (2025). Leadless Pacing in Peri-Procedural Settings. Eur. Heart J. Suppl..

[63] Palmisano P., Iacopino S., De Vivo S., D’Agostino C., Tomasi L., Startari U., Ziacchi M., Pisanò E.C.L., Santobuono V.E., Caccavo V.P. (2022). Leadless Transcatheter Pacemaker: Indications, Implantation Technique and Peri-Procedural Patient Management in the Italian Clinical Practice. Int. J. Cardiol..

[64] Oliveira V.M.R., Rivera A., Oliveira I.C., de Sousa A.M., Nishikubo M.E.P., Serpa F., da Silva Menezes Junior A. (2024). The Effectiveness and Safety of Leadless Pacemakers: An Updated Meta-Analysis. Curr. Cardiol. Rep..

[65] Khan M.Z., Nassar S., Nguyen A., Khan M.U., Sattar Y., Alruwaili W., Gonuguntla K., Mazek H., Asad Z.U.A., Agarwal S. (2024). Contemporary Trends of Leadless Pacemaker Implantation in the United States. J. Cardiovasc. Electrophysiol..

[66] Miller J.D., Yousuf O., Berger R.D. (2015). The Implantable Cardioverter-Defibrillator: An Update. Trends Cardiovasc. Med..

[67] Santobuono V.E., Favale S., D’Onofrio A., Manzo M., Calò L., Bertini M., Savarese G., Santini L., Dello Russo A., Lavalle C. (2023). Performance of a Multisensor Implantable Defibrillator Algorithm for Heart Failure Monitoring Related to Co-Morbidities. ESC Heart Fail..

[68] Stringer B., MacLeod L., Kaldas F., Krishnasamy G., Khan H.R. (2025). Implantable Cardiac Defibrillator Outcomes in Octogenarians. J. Arrhythm..

[69] Maron B.J., Estes N.A.M., Rowin E.J., Maron M.S., Reynolds M.R. (2023). Development of the Implantable Cardioverter-Defibrillator: JACC Historical Breakthroughs in Perspective. J. Am. Coll. Cardiol..

[70] Guarracini F., Preda A., Bonvicini E., Coser A., Martin M., Quintarelli S., Gigli L., Baroni M., Vargiu S., Varrenti M. (2023). Subcutaneous Implantable Cardioverter Defibrillator: A Contemporary Overview. Life.

[71] Wolf S., Götz G., Wernly B., Wild C. (2023). Subcutaneous Implantable Cardioverter-Defibrillator: A Systematic Review of Comparative Effectiveness and Safety. ESC Heart Fail..

[72] Kaya E., Rassaf T., Wakili R. (2019). Subcutaneous ICD: Current Standards and Future Perspective. IJC Heart Vasc..

[73] Crozier I., O’Donnell D., Boersma L., Murgatroyd F., Manlucu J., Knight B.P., Birgersdotter-Green U.M., Leclercq C., Thompson A., Sawchuk R. (2021). The Extravascular Implantable Cardioverter-Defibrillator: The Pivotal Study Plan. J. Cardiovasc. Electrophysiol..

[74] Garibaldi S., Aimo A., Concistrè G., Nesti M., Panchetti L., Startari U., Mirizzi G., Santoro G., Piacenti M., Rossi A. (2025). Extravascular Implantable Cardioverter-Defibrillator Implantation in a Teenager with Brugada Syndrome and Recurrent Inappropriate Shocks of Subcutaneous Implantable Cardioverter-Defibrillator. HeartRhythm Case Rep..

[75] Yuecel G., Gaasch L., Kodeih A., Hetjens S., Yazdani B., Pfleger S., Duerschmied D., Abraham W.T., Akin I., Kuschyk J. (2025). Device-Therapy in Chronic Heart Failure: Cardiac Contractility Modulation versus Cardiac Resynchronization Therapy. ESC Heart Fail..

[76] Laina A., Antoniou C.K., Tsiachris D., Kordalis A., Arsenos P., Doundoulakis I., Dilaveris P., Xintarakou A., Xydis P., Soulaidopoulos S. (2025). CRT-D or CRT-P: When There Is a Dilemma and How to Solve It. J. Clin. Med..

[77] Tolu-Akinnawo O., Akhtar N., Zalavadia N., Guglin M. (2025). CardioMEMS Heart Failure System: An Up-to-Date Review. Cureus.

[78] Zile M.R., Mehra M.R., Ducharme A., Sears S.F., Desai A.S., Maisel A., Paul S., Smart F., Grafton G., Kumar S. (2022). Hemodynamically-Guided Management of Heart Failure Across the Ejection Fraction Spectrum: The GUIDE-HF Trial. JACC Heart Fail..

[79] Givertz M.M., Stevenson L.W., Costanzo M.R., Bourge R.C., Bauman J.G., Ginn G., Abraham W.T. (2017). Pulmonary Artery Pressure-Guided Management of Patients with Heart Failure and Reduced Ejection Fraction. J. Am. Coll. Cardiol..

[80] McDonagh T.A., Metra M., Adamo M., Baumbach A., Böhm M., Burri H., Čelutkiene J., Chioncel O., Cleland J.G.F., Coats A.J.S. (2021). 2021 ESC Guidelines for the Diagnosis and Treatment of Acute and Chronic Heart Failure. Eur. Heart J..

[81] Guichard J.L., Cowger J.A., Chaparro S.V., Kiernan M.S., Mullens W., Mahr C., Mullin C., Forouzan O., Hiivala N.J., Sauerland A. (2023). Rationale and Design of the Proactive-HF Trial for Managing Patients With NYHA Class III Heart Failure by Using the Combined Cordella Pulmonary Artery Sensor and the Cordella Heart Failure System. J. Card. Fail..

[82] Sharif F., Rosenkranz S., Bartunek J., Kempf T., Aßmus B., Mahon N.G., Hiivala N.J., Mullens W. (2024). Twelve-Month Follow-up Results from the SIRONA 2 Clinical Trial. ESC Heart Fail..

[83] Bayes-Genis A., Pagnesi M., Codina P., Abraham W.T., Amir O., de Boer R.A., Brugts J.J., Chioncel O., Gustafsson F., Lindenfeld J.A. (2025). Remote Pulmonary Artery Pressure-Guided Management of Patients with Heart Failure: A Clinical Consensus Statement of the Heart Failure Association (HFA) of the ESC. Eur. J. Heart Fail..

[84] Perl L., Meerkin D., D’amario D., Avraham B.B., Gal T.B., Weitsman T., Hasin T., Ince H., Feickert S., D’ancona G. (2022). The V-LAP System for Remote Left Atrial Pressure Monitoring of Patients With Heart Failure: Remote Left Atrial Pressure Monitoring. J. Card. Fail..

[85] Perl L., Ben Avraham B., Vaknin-Assa H., Ben Gal T., Kornowski R. (2020). A Rise in Left Atrial Pressure Detected by the V-LAP^TM^ System for Patients with Heart Failure during the Coronavirus Disease 2019 Pandemic. ESC Heart Fail..

[86] Mutirangura P., Patel D., Akram H., Hughes A., Arriola-Montenegro J., Koukousaki D., Money J., Kosmopoulos M., Meyer M., Harata M. (2025). Application of Telemedicine in the Management of Cardiovascular Diseases: A Focus on Heart Failure. Rev. Cardiovasc. Med..

[87] Meerkin D., Perl L., Hasin T., Petriashvili S., Kurashvili L., Metreveli M., Ince H., Feickert S., Habib M., Caspi O. (2024). Physician-Directed Patient Self-Management in Heart Failure Using Left Atrial Pressure: Interim Insights from the VECTOR-HF I and IIa Studies. Eur. J. Heart Fail..

[88] Maimaiti M., Zhu H., Zeng H. (2025). Applications of Ballistocardiogram in the Diagnosis of Coronary Heart Disease: Systematic Review. JMIR Cardio.

[89] Feng J., Huang W.F., Jiang J., Wang Y., Zhang X., Li Q., Jiao X. (2023). Non-Invasive Monitoring of Cardiac Function through Ballistocardiogram: An Algorithm Integrating Short-Time Fourier Transform and Ensemble Empirical Mode Decomposition. Front. Physiol..

[90] Zhan J., Li Z., Wu X., Zhang C., Zhao T., Chen K., Lu Z. (2025). A Multi-Pathology Ballistocardiogram Dataset for Cardiac Function Monitoring and Arrhythmia Assessment. Sci. Data.

[91] Centracchio J., Parlato S., Esposito D., Bifulco P., Andreozzi E. (2023). ECG-Free Heartbeat Detection in Seismocardiography Signals via Template Matching. Sensors.

[92] Rahman M.M., Cook J., Taebi A. (2023). Non-Contact Heart Vibration Measurement Using Computer Vision-Based Seismocardiography. Sci. Rep..

[93] Agam A., Agam A., Korsgaard E., Yding T., Kristensen C.B., Mogelvang R., Kragholm K., Grønn Emerk K.J., Søgaard P., Schmidt S.E. (2025). Evaluation of Seismocardiography in Detecting Pre-Load Changes and Cardiovascular Disease: A Comparative Study with Transthoracic Echocardiography. Eur. Heart J.—Digit. Health.

[94] Otake Y., Kobayashi T., Hakozaki Y., Matsui T. (2021). Non-Contact Heart Rate Variability Monitoring Using Doppler Radars Located beneath Bed Mattress: A Case Report. Eur. Heart J. Case Rep..

[95] Lu Z., Chen J., Zhang D., Wang H., Huang P., Zhou F., Hu Y., Sun Q., Gao M., Chen Y. (2026). Contactless 12-Lead Electrocardiogram via Deep Computational Radar. npj Biomed. Innov..

[96] Ota T., Okusa K. (2024). Model-Based Estimation of Heart Movements Using Microwave Doppler Radar Sensor. J. Physiol. Anthropol..

[97] Antolinos E., García-Rial F., Hernández C., Montesano D., Godino-Llorente J.I., Grajal J. (2020). Cardiopulmonary Activity Monitoring Using Millimeterwave Radars. Remote Sens..

[98] van Vliet M., Aalberts J.J.J., Hamelinck C., Hauer A.D., Hoftijzer D., Monnink S.H.J., Schipper J.C., Constandse J.C., Peters N.S., Lip G.Y.H. (2025). Assessment of Photoplethysmography-Based Blood Pressure Determinations during Long-Term and Short-Term Remote Cardiac Monitoring: The RECAMO Study. Eur. Heart J.—Digit. Health.

[99] Väliaho E.S., Lipponen J.A., Kuoppa P., Martikainen T.J., Jäntti H., Rissanen T.T., Castrén M., Halonen J., Tarvainen M.P., Laitinen T.M. (2022). Continuous 24-h Photoplethysmogram Monitoring Enables Detection of Atrial Fibrillation. Front. Physiol..

[100] Hereijgers M.J.M., van der Velden R.M.J., Reinders L.G., Weerts Z., Kuijpers S., Luermans J., Chaldoupi S.M., Vernooy K., Hermans A.N.L., Posthuma R. (2025). Feasibility and Diagnostic Yield of a Photoplethysmography-Based Arrhythmia Screening and Integrated Management Pathway in a COPD Outpatient Clinic. ERJ Open Res..

[101] Butler J., Brown M., Prokocimer P., Humphries A.C., Pope S., Wright O., Su J., Elnawasany O., Muresan B. (2025). The Role of Cardiac Acoustic Biomarkers in Monitoring Patients with Heart Failure: A Systematic Literature Review. ESC Heart Fail..

[102] Damera V.K., Cheripelli R., Putta N., Sirisha G., Kalavala D. (2025). Enhancing Remote Patient Monitoring with AI-Driven IoMT and Cloud Computing Technologies. Sci. Rep..

[103] Saner H., Knobel S.E.J., Schuetz N., Nef T. (2020). Contact-Free Sensor Signals as a New Digital Biomarker for Cardiovascular Disease: Chances and Challenges. Eur. Heart J.—Digit. Health.

[104] Mariani M.V., Lavalle C., Forleo G.B., Della Rocca D.G., Martino A., Panuccio M., Fagagnini A., Rebecchi M., Calò L., Santini L. (2023). HeartLogic^TM^: Real-World Data—Efficiency, Resource Consumption, and Workflow Optimization. Eur. Heart J. Suppl..

[105] Rahul J., Sharma L.D. (2025). Advancements in AI for Cardiac Arrhythmia Detection: A Comprehensive Overview. Comput. Sci. Rev..

[106] Reinhardt A., Ventura R. (2023). Remote Monitoring of Cardiac Implantable Electronic Devices: What Is the Evidence?. Curr. Heart Fail. Rep..

[107] Hassanpour A., Yang B. (2025). Contactless Vital Sign Monitoring: A Review Towards Multi-Modal Multi-Task Approaches. Sensors.

[108] Bartusik-Aebisher D., Rogóż K., Aebisher D. (2025). Artificial Intelligence and ECG: A New Frontier in Cardiac Diagnostics and Prevention. Biomedicines.

[109] Zavaleta Cavero J.A., Flores Pérez M.A., De Moura Mendoza J., Paricanaza Bravo R., Huané L.C. (2025). SUNQUI: Ambulatory Arrhythmia Monitoring Device Based on Artificial Intelligence Pre-Diagnosis in Electrocardiography. Proceedings of the 2025 47th Annual International Conference of the IEEE Engineering in Medicine and Biology Society (EMBC), Copenhagen, Denmark, 14 July 2025.

[110] Liu L., Fang W. (2021). Artificial Intelligence-Based Remote Electrocardiogram Monitoring Improves the Diagnosis of Arrhythmias and Reduces Morbidity Caused by Fatal Arrhythmias. J. Prev. Med. Healthc..

[111] Ghasemieh A., Lloyed A., Bahrami P., Vajar P., Kashef R. (2023). A Novel Machine Learning Model with Stacking Ensemble Learner for Predicting Emergency Readmission of Heart-Disease Patients. Decis. Anal. J..

[112] Varma N., Cygankiewicz I., Turakhia M.P., Heidbuchel H., Hu Y., Chen L.Y., Couderc J.P., Cronin E.M., Estep J.D., Grieten L. (2021). 2021 ISHNE/HRS/EHRA/APHRS Collaborative Statement on MHealth in Arrhythmia Management: Digital Medical Tools for Heart Rhythm Professionals: From the International Society for Holter and Noninvasive Electrocardiology/Heart Rhythm Society/European Heart Rhythm Association/Asia Pacific Heart Rhythm Society. Cardiovasc. Digit. Health J..

[113] Boehmer J.P., Hariharan R., Devecchi F.G., Smith A.L., Molon G., Capucci A., An Q., Averina V., Stolen C.M., Thakur P.H. (2017). A Multisensor Algorithm Predicts Heart Failure Events in Patients With Implanted Devices. JACC Heart Fail..

[114] Cricco R., Segreti A., Ferro A., Beato S., Castaldo G., Ciancio M., Sacco F.M., Pennazza G., Ussia G.P., Grigioni F. (2025). Advancements in Sensor Technology for Monitoring and Management of Chronic Coronary Syndrome. Sensors.

[115] Mohd Faizal A.S., Thevarajah T.M., Khor S.M., Chang S.W. (2021). A Review of Risk Prediction Models in Cardiovascular Disease: Conventional Approach vs. Artificial Intelligent Approach. Comput. Methods Programs Biomed..

[116] Charlton P.H., Kyriacou P.A., Mant J., Marozas V., Chowienczyk P., Alastruey J. (2022). Wearable Photoplethysmography for Cardiovascular Monitoring. Proc. IEEE.

[117] Chen L.C., Hung K.H., Tseng Y.J., Wang H.Y., Lu T.M., Huang W.C., Tsao Y. (2024). Self-Supervised Learning-Based General Laboratory Progress Pretrained Model for Cardiovascular Event Detection. IEEE J. Transl. Eng. Health Med..

[118] Svandova K., Smutny Z. (2024). Internet of Medical Things Security Frameworks for Risk Assessment and Management: A Scoping Review. J. Multidiscip. Healthc..

[119] Lyon A., Mincholé A., Martínez J.P., Laguna P., Rodriguez B. (2018). Computational Techniques for ECG Analysis and Interpretation in Light of Their Contribution to Medical Advances. J. R. Soc. Interface.

[120] Theerthagiri P., Vidya J. (2022). Cardiovascular Disease Prediction Using Recursive Feature Elimination and Gradient Boosting Classification Techniques. Expert Syst..

[121] Vandenberk B., Chew D.S., Prasana D., Gupta S., Exner D.V. (2023). Successes and Challenges of Artificial Intelligence in Cardiology. Front. Digit. Health.

[122] Chung C.T., Lee S., King E., Liu T., Armoundas A.A., Bazoukis G., Tse G. (2022). Clinical Significance, Challenges and Limitations in Using Artificial Intelligence for Electrocardiography-Based Diagnosis. Int. J. Arrhythmia.

[123] Lewin S., Chetty R., Ihdayhid A.R., Dwivedi G. (2024). Ethical Challenges and Opportunities in Applying Artificial Intelligence to Cardiovascular Medicine. Can. J. Cardiol..

[124] Patel D., Chetarajupalli C., Khan S., Khan S., Patel T., Joshua S., Millis R.M. (2025). A Narrative Review on Ethical Considerations and Challenges in AI-Driven Cardiology. Ann. Med. Surg..

[125] Ebad S.A., Alhashmi A., Amara M., Miled A.B., Saqib M. (2025). Artificial Intelligence-Based Software as a Medical Device (AI-SaMD): A Systematic Review. Healthcare.

